# Coronary flow reserve in adults with systemic right or single ventricles

**DOI:** 10.1186/1532-429X-18-S1-P173

**Published:** 2016-01-27

**Authors:** Laura Olivieri, Li-Yueh Hsu, Anitha John, Karen Kuehl, Andrew E Arai

**Affiliations:** 1 Children's National Medical Center, Washington, DC USA; 2 National Heart, Lung and Blood Institute, Bethesda, MD USA

## Background

Late failure of systemic right and single ventricles is difficult to predict. Approximately 1/3 of subjects with congenitally corrected transposition of the great arteries (ccTGA) have congestive heart failure by the fifth decade and 2/3 of subjects with ccTGA and significant associated defects have congestive heart failure by the age of 45 years. Perfusion defects have been identified at rest in 55 % of subjects with systemic right ventricles by dipyridamole sestamibi and with exercise in 45 %, which are not due to endovascular obstructive coronary disease. Subjects with perfusion defects typically have worse right ventricular function, but not always. It may be tied to coronary flow reserve and coronary mismatch in these hypertrophied ventricles. The objective of this study was to assess the technical feasibility of stress perfusion imaging by CMR and calculate coronary flow reserve in adults with systemic right or single ventricles.

## Methods

With IRB approval and informed consent, 14 adult patients with either a systemic right ventricle or a single ventricle underwent CMR with vasodilator stress perfusion imaging using adenosine (140 mcg/kg/min). Data collection included stress perfusion imaging, measurement of cardiac volume metrics and rest perfusion imaging at least 20 minutes after adenosine. Global myocardial blood flow (MBF) in ml/min/g and coronary flow reserve (CFR) were quantified using a fully quantitative model constrained deconvolution. Subjects underwent additional data collection, including lab work, 6-minute walk test and ECG.

## Results

**Figure 1:**
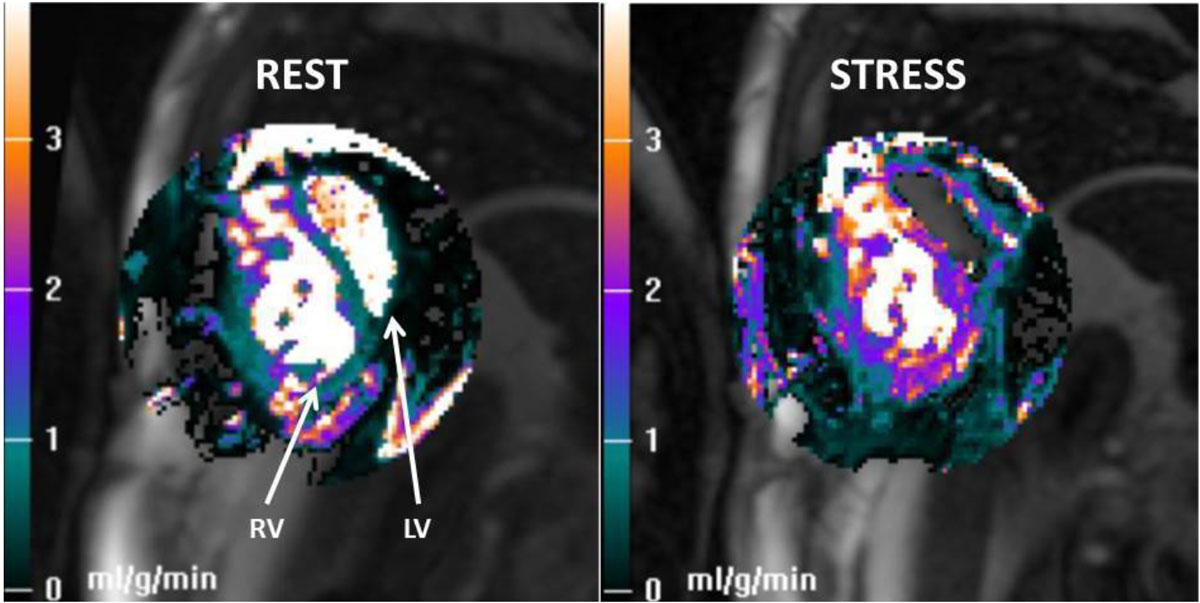
**Quantitative pixel maps showing myocardial blood flow values for a patient with D-TGA s/p Mustard at rest (left) and during adenosine stress (right)**.

Table 1 summarizes patient characteristics and salient testing results. Two of 14 perfusion studies were not quanitifiable; one due to an incomplete first pass of contrast likely related to low EF, one had technical issues during imaging. Of the remaining 12 subjects, 10 had a measureable change in myocardial blood flow, and 2 did not. Stress measurements of myocardial blood flow were lower in most patients than established MBF values in normal ventricles. Figure 1 demonstrates the global myocardial blood flow at rest and during stress of a patient with high CFR.

**Table 1:** Characteristics and measured myocardial blood flow and coronary flow reserve of 12 included subjects.

Diagnosis	Ventricular morphology	EDVi (ml/m2)	EF	Stress MBF (ml/g/min)	Rest MBF (ml/g/min)	Flow Reserve (ml/g/min)
DTGA Mustard	RV	161.2	40%	1.55	1.05	0.5
DTGA Mustard	RV	107.1	45%	1.11	0.83	0.28
ccTGA {S,L,L}	RV	205	54%	0.94	0.92	0.02
PA-IVS Fontan	LV	56.4	58%	0.77	0.73	0.04
ccTGA {S,L,L}	RV	115.5	50%	1.68	1.10	0.58
DTGA Mustard	RV	111.7	53%	2.07	1.14	0.93
DILV, {S,L,L} Fontan	LV	104.1	63%	1.07	0.83	0.24
DTGA Senning	RV	124.7	45%	2.05	1.28	0.77
HLHS Fontan	RV	95.5	65%	1.7	1.23	0.47
ccTGA {S,L,L}	RV	90.6	50%	2.13	0.9	1.23
ccTGA, VSD s/p closure	RV	153.9	50%	2.99	1.26	1.73
DILV, Fontan	LV	57	68%	1.64	0.76	0.88

(ccTGA congenitally corrected Transposition of the great arteries, DTGA D-transposition of the great arteries, PA-IVS pulmonary atresia with intact ventricular septum, DILV double inlet left ventricle, HLHS hypoplastic left heart syndrome, VSD ventricular septal defect, EDVi end-diastolic volume indexed to body surface area, EF ejection fraction, MBF myocardial blood flow).

## Conclusions

Myocardial blood flow and coronary flow reserve are quantifiable with current techniques regardless of ventricular morphology. This technique shows promise as a potential method of risk stratification of this population.

